# Supplementary material for: Thymus vulgaris L. Essential Oil Solid Formulation: Chemical Profile and Spasmolytic and Antimicrobial Effects
Source: Biomolecules. 2020 Jun 4;10(6):860. doi: 10.3390/biom10060860 (PMC7356897; doi:10.3390/biom10060860)
Supplement: Supplementary file 1 [file biomolecules-10-00860-s001.pdf]

Supporting Material

Thymus vulgaris L. essential oil solid formulation: chemical profile, spasmolytic and antimicrobial effects

Matteo Micucci[1#], Michele Protti[2#], Rita Aldini[1], Maria Frosini[3], Ivan Corazza[4], Carla Marzetti[5], Laura Beatrice Mattioli[1], Gabriella Tocci[5], Alberto Chiarini[1,6], Laura Mercolini[2*], Roberta Budriesi[1*]

1 Department of Pharmacy and Biotechnology, Nutraceutical Lab, Alma Mater Studiorum - University of Bologna, 40126 Bologna, Italy; matteo.micucci2@unibo.it (M.M.); rita.aldini@unibo.it (R.A.); laubea91@gmail.com (L.B.M.); alberto.chiarini@unibo.it (A.C.)

2 Department of Pharmacy and Biotechnology, Research group of Pharmaco-Toxicological Analysis (PTA Lab), Alma Mater Studiorum - University of Bologna, 40126 Bologna, Italy; michele.protti2@unibo.it (M.P.)

3 Department of Life Sciences, Vita, University of Siena, 53100 Siena, Italy; maria.frosini@unisi.it (M.F.)

4 Department of Experimental, Diagnostic and Specialty Medicine - DIMES, Alma Mater Studiorum - University of Bologna, 40138 Bologna, Italy; ivan.corazza@unibo.it (I.C.)

5 Valsambro S.r.l., Bologna, Italy; carla.marzetti@valsambro.it (C.M.); gabriella.tocci@valsambro.it (G.T.)

6 GVM Care & Research, Lugo, Ravenna, Italy

* Correspondence: roberta.budriesi@unibo.it; Tel.: +39 051 20 9 9737 (R.B.); laura.mercolini@unibo.it; Tel.: +39 051 20 9 9726 (L.M.)

\# Contributed equally to the work.

S1. LC-MS/MS conditions

The LC-MS/MS analyses were performed on a Waters Alliance e2685 chromatographic system with autosampler coupled to a Waters Micromass Quattro Micro triple-quadrupole mass analyser through an electrospray ionisation source operating in negative ionisation mode (ESI-). Data were processed with Waters MassLynx 4.1 software. Separations were obtained on a Restek Ultra AQ C18 column (50 x 2.1 mm ID, 3 μm) equipped with a guard column and kept at room temperature. The mobile phase was a mixture of 0.2 formic acid in water (component A) and 0.2% formic acid in ACN (component B) at a constant flow rate of 0.3 mL/min under the following composition gradient program: 0-1 min, A:B = 70:30; 3 min, A:B = 30:70; 3-6 min, A:B = 30:70; 8 min, A:B = 70:30; 8-10 min, A:B = 70:30. Injection volume was 10 μL. Multiple reaction monitoring (MRM) transitions were acquired in negative ionisation mode. Being positional isomers, thymol and carvacrol were determined using the same set of transitions and identified by their retention times. MRM transitions were as follows: m/z 149.23 → 133.1, m/z 149.23 → 106.3, m/z 149.23 → 91.1, being the former one exploited for quantitative purposes and the other two monitored for qualitative confirmation. Other optimised MS parameters were as follows: ion source voltage -3.5 kV, cone voltage -26 V, collision energy -33 V, ion source temperature 100 °C, desolvation temperature 140°C, desolvation gas flow 750 L/h, nitrogen was used as desolvation gas, argon as collision gas.

S2. Details for in vitro studies

Gastric fundus. The stomach was removed, opened along the mesentery of the greater curvature, and rinsed with Krebs bicarbonate-buffered solution of the following

composition (mM): NaCl 120, KCl 4.6, $CaCl_2$ 2.5, $MgCl_2$ 1.2, $NaH_2PO_4{\bullet}2H_2O$ 1.2, $NaHCO_3$ 22, and glucose 11.5; maintained at 37°C as previously described. [SR1]

Ileum. The terminal portion of ileum (3–4 cm near the ileo-caecal junction) was cleaned, and segments 2–3 cm long of ileum were set up under 1 g tension at 37 °C in organ baths containing Tyrode solution of the following composition (mM): NaCl, 118; KCl, 4.75; $CaCl_2$, 2.54; $MgSO_4{\bullet}7H_2O$, 1.20; $KH_2PO_4{\bullet}2H_2O$, 1.19; $NaHCO_3$ 25; glucose 11. The two segments obtained (2–3 cm) were set up under 1 g tension in the longitudinal direction along the intestinal wall. Tissue were allowed to equilibrate for at list 30 min during which time the bathing solution was changed every 10 min. The two segments obtained (2–3 cm) were set up under 1 g tension in the longitudinal direction along the intestinal wall.

Proximal colon. Starting approximately 1 cm distal from the caecocolonic junction, two segments of about 1 cm of the guinea-pig proximal colon was cut. The proximal colon was cleaned by rinsing it with De Jalon solution of the following composition (mM): NaCl, 155; KCl, 5.6; $CaCl_2$, 0.5; $NaHCO_3$, 6.0; glucose, 2.8; and the mesenteric tissue was removed. The two segments were suspended in organ baths containing gassed warm de Jalon solution under a load of 1 g maintained at 37°C. Tension changes in longitudinal muscle length were recorded. Tissue were allowed to equilibrate for at list 30 min during which time the bathing solution was changed every 10 min.

*Gallbladde*r. The gallbladder was opened and washed in Krebs solution of the following composition (mM): NaCl 118, KCl 4.7, $CaCl_2$ 2.5, $MgSO_4{\bullet}7H_2O$ 1.2, $KH_2PO_4{\bullet}2H_2O$ 1.2, $NaHCO_3$ 24.9, and glucose 11.1. The strips of gallbladder were mounted in organ baths containing Krebs solution (15 mL) maintained at 37°C. [SR2]

For explanatory purposes we attach a picture of our data recording apparatus (see above).

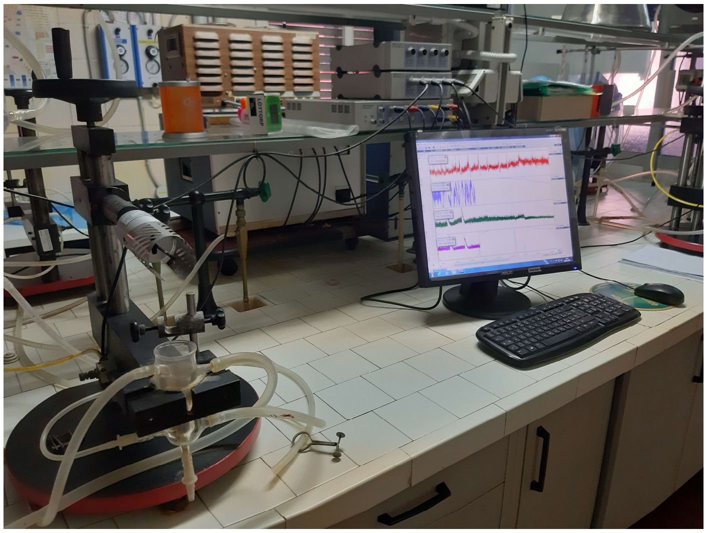

S3. Details for antibacterial activity

Potential antibacterial activity of EO and SEO was tested against Gram-positive *Staphylococcus aureus* (ATCC 25923 KS2) and *Streptococcus pyogenes* (ATCC 19615); Gram-negative *Escherichia coli* (ATCC 700728), *Salmonella Thyphimurium* (ATCC 14028), *Pseudomonas Aeruginosa* (ATCC 27853), *Bifidobacterium Breve* (ATCC 15700), *Lactobacillus Fermentum* (ATCC 9338) and yeast *Candida albicans* (ATCC 14053). The standard anti-microbic agent was Cyprofloaxacin. All strains were subjected to antimicrobial susceptibility test using Kirby-Bauer disc diffusion method as described by National Committee for Clinical Laboratory Standards Guidelines, 1993 (NCCLS). The Minimal Inhibitory Concentrations (MICs) values were proposed for seven microorganism determined by the microdilution method. [SR3] Data were evaluated using the IBM SPSS software program (version 19; IBM SPSS Inc., IL USA). All samples and control groups were compared at the 95% confidence interval. Details about cultures and anti-microorganism assays were previously described. [SR4] DMSO (10%) was used as solvent for diluition.

S4. Analytical method development and validation

In order to effectively detect and quantify the selected panel of target compounds setting up the most suitable analytical methodologies, different C8 and C18 sorbents were tested as the stationary phases, coupled to mixtures of acidic buffers for CEC (pH 3.0-6.5) or formic acid concentrations for LC (0.05%-0.5%) and organic solvents (methanol and acetonitrile) to be used as the mobile phase. DAD detector allowed UV spectrum screening, in order to verify the best wavelengths able to grant good signal-to-noise ratios, while for MS/MS, parameters were carefully tuned on standard solutions to obtain the highest response. By applying the optimised conditions, peak resolution and chromatographic run times were satisfactory, thus the methods were fully validated in terms of linearity, precision and accuracy. For CEC-DAD, method sensitivity was found to be between 0.6 μg/mL and 1.5 μg/mL in terms of LOD and between 2 μg/mL and 5 μg/mL in terms of LOQ, respectively. Linearity was tested in the 5-200 μg/mL range for all the analytes, by injecting in triplicate standard solutions of the analytes at known concentrations and setting up linearity curves by plotting peak areas versus nominal concentrations using the least-square method ($r^2 \geq 0.9991$). For LC-MS/MS, LOQ and LOD values were 20 ng/mL and 6 ng/mL respectively, for both thymol and carvacrol, while method was linear in the 20-1000 ng/mL range ($r^2 \geq 0.9997$). Method precision was tested in terms of percentage relative standard deviation (RSD%) by injecting five replicates of three known concentrations of each analyte representative of the respective linearity ranges. Satisfactory results were obtained for both CEC-DAD and LC-MS/MS, with RSD values always < 5.7% and < 4.5%, respectively (< 4.1% for IS). For method accuracy, known amounts of the analytes at three levels of each calibration curve were added to samples whose analyte content was already assessed, then the spiked samples were analysed in triplicate to obtain the mean recovery (%). Satisfactory results were obtained, being accuracy always higher than 85% for CEC-DAD and 91% for LC-MS/MS. Complete CEC-DAD and LC-MS/MS method validation results are reported in Table S1.

Table S1. CEC-DAD and LC-MS/MS method validation results.

CEC-DAD								
Analyte	Linearity range (ng/mL)	r^2	LOD (µg/mL)	LOQ (µg/mL)	Concentration level (µg/mL)	Precision (RSD%)		Accuracy ± SD (%)
						Intraday	Interday	
p-Cymene	5-200	0.9993	1.2	3.5	5.0	4.9	5.1	86 ± 2
					100	4.5	4.8	88 ± 3
					200	4.4	4.6	90 ± 2
α-Terpinene	5-200	0.9994	0.7	2.2	5.0	5.0	5.3	87 ± 3
					100	4.9	5.0	90 ± 4
					200	4.3	4.5	91 ± 2
γ-Terpinene	5-200	0.9991	1.5	5.0	5.0	5.3	5.6	86 ± 4
					100	5.2	5.4	87 ± 3
					200	4.8	4.9	88 ± 3
β-Myrcene	5-200	0.9994	1.0	3.0	5.0	5.2	5.4	91 ± 3
					100	5.0	5.2	89 ± 5
					200	4.6	4.7	88 ± 2
Limonene	5-200	0.9993	0.8	2.5	5.0	4.7	4.9	86 ± 2
					100	4.1	4.4	89 ± 1
					200	4.0	4.2	90 ± 2
β-Pinene	5-200	0.9995	0.6	2.0	5.0	4.7	4.8	89 ± 2
					100	4.2	4.5	91 ± 1
					200	3.9	4.1	94 ± 3
Thymol	5-200	0.9996	0.6	2.0	5.0	4.7	4.8	91 ± 2
					100	4.1	4.3	92 ± 3
					200	3.9	4.1	95 ± 2
Carvacrol	5-200	0.9997	0.7	2.2	5.0	5.0	5.2	90 ± 3
					100	4.5	4.7	93 ± 2
					200	4.1	4.5	96 ± 2
Linalool	5-200	0.9995	0.6	2.0	5.0	5.0	5.3	89 ± 4
					100	4.9	5.1	91 ± 3
					200	4.6	4.9	92 ± 2
α-Terpineol	5-200	0.9993	0.6	2.0	5.0	4.7	4.9	87 ± 4
					100	4.1	4.4	88 ± 3
					200	3.8	4.0	91 ± 3
Borneol	5-200	0.9994	0.7	2.2	5.0	5.0	5.1	88 ± 3
					100	4.6	4.9	90 ± 4
					200	4.4	4.5	91 ± 1
β-Cariophyllene	5-200	0.9991	1.2	3.5	5.0	5.3	5.6	86 ± 5
					100	4.8	5.3	88 ± 3
					200	4.8	5.0	89 ± 2
IS					100	3.7	4.0	95 ± 1

LC-MS/MS								
Analyte	Linearity range (ng/mL)	r^2	LOD (ng/nL)	LOQ (ng/nL)	Concentration level (ng/mL)	Precision (RSD%)		Accuracy ± SD (%)
						Intraday	Interday	
Thymol	20-1000	0.9998	6	20	20	4.0	3.9	94 ± 2
					100	3.4	3.1	96 ± 2
					1000	3.2	2.9	98 ± 1
Carvacrol	20-1000	0.9997	6	20	20	4.2	4.4	92 ± 2
					100	3.6	4.0	94 ± 1
					1000	3.3	3.7	94 ± 1

Figure S5. Effects of EO, SEO, excipients and OB on ileum Spontaneous contraction rates (FFT). Absolute band powers of control and after addition of each concentration observed in the same experiment (FFT).

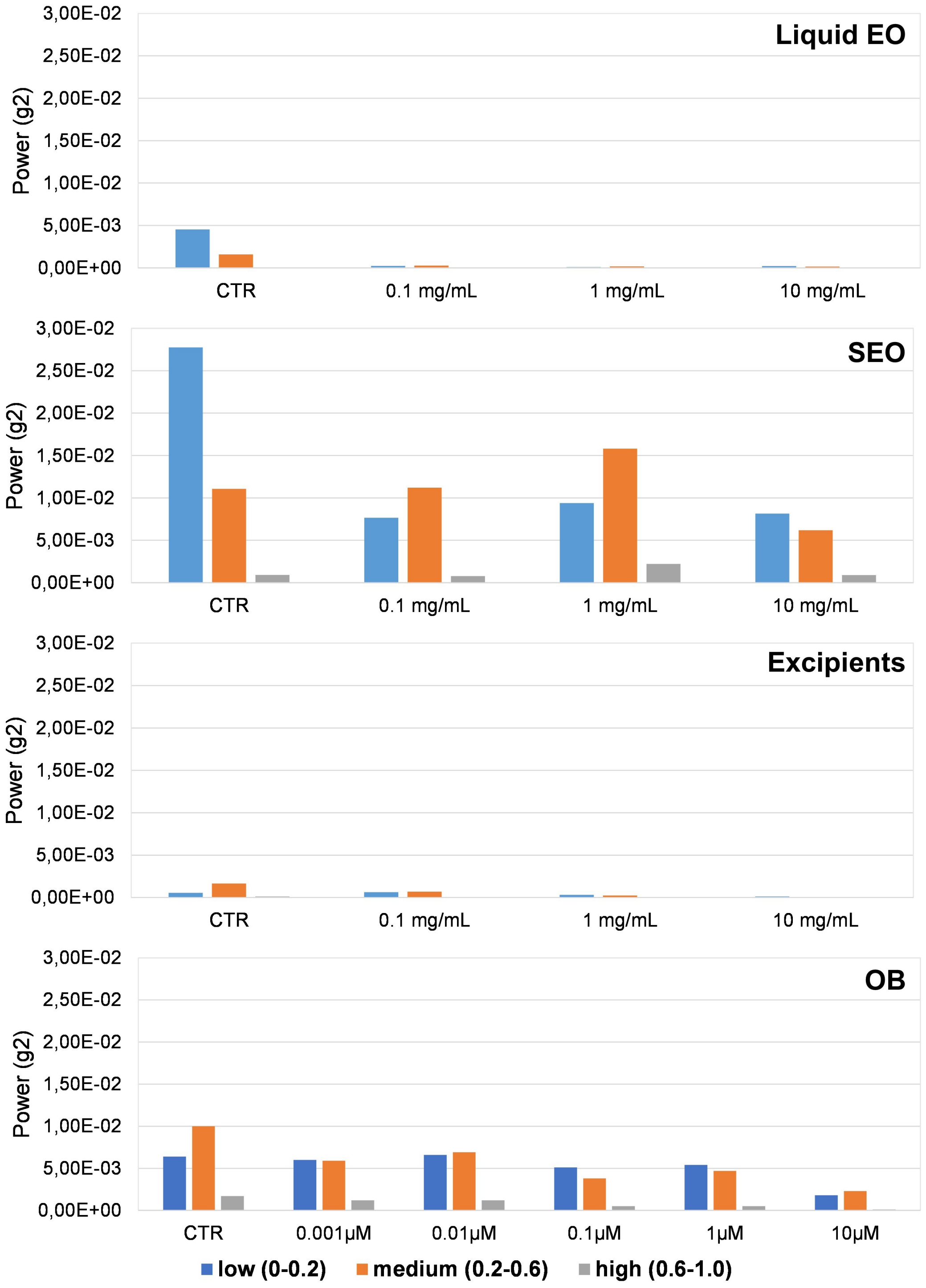

Figure S6. Effects of EO, SEO, excipients and OB on colon Spontaneous contraction rates (FFT). Absolute band powers of control and after addition of each concentration observed in the same experiment (FFT).

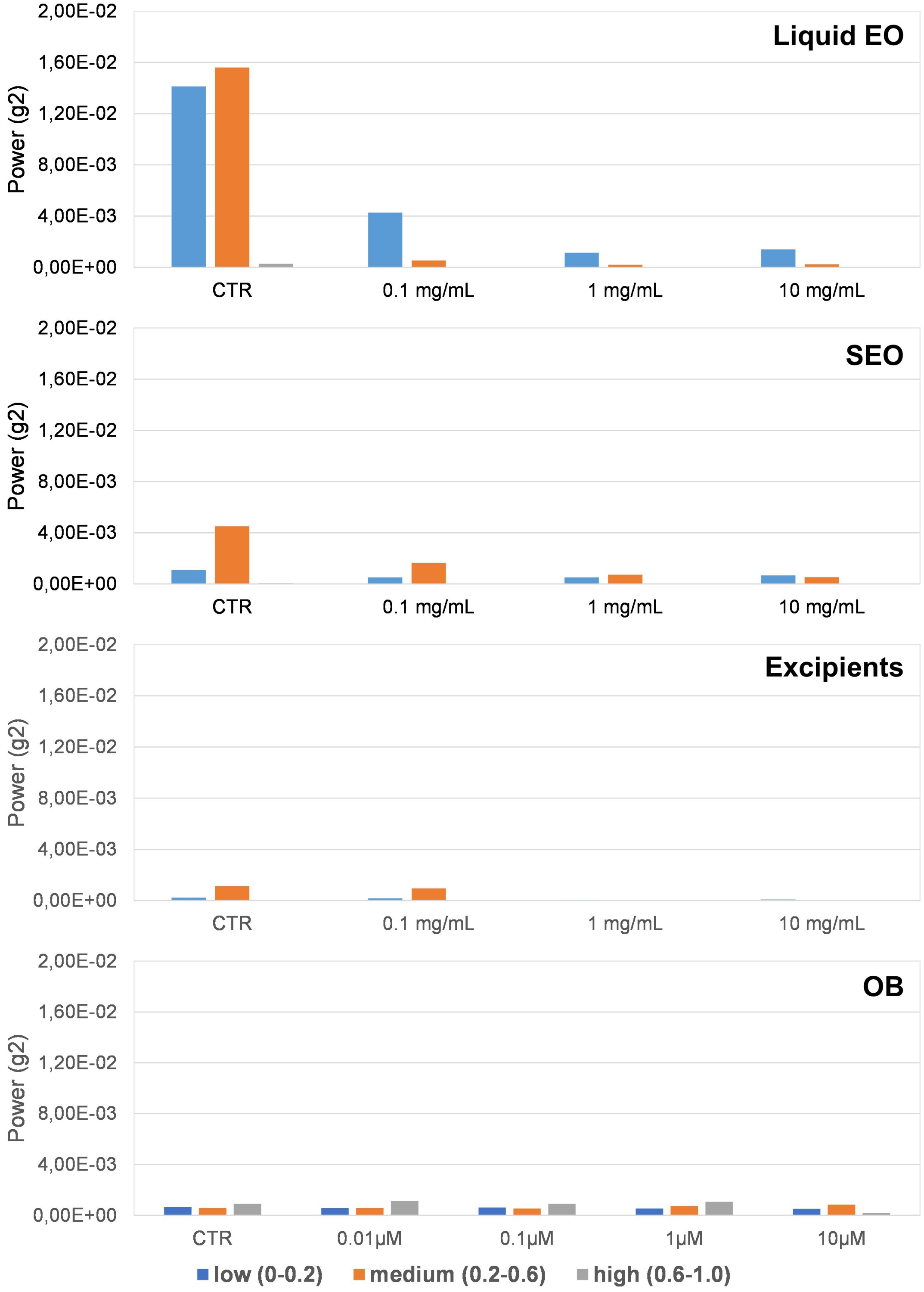

Figure S7. Effects of EO, SEO, excipients and OB on gallbladder Spontaneous contraction rates (FFT). Absolute band powers of control and after addition of each concentration observed in the same experiment (FFT).

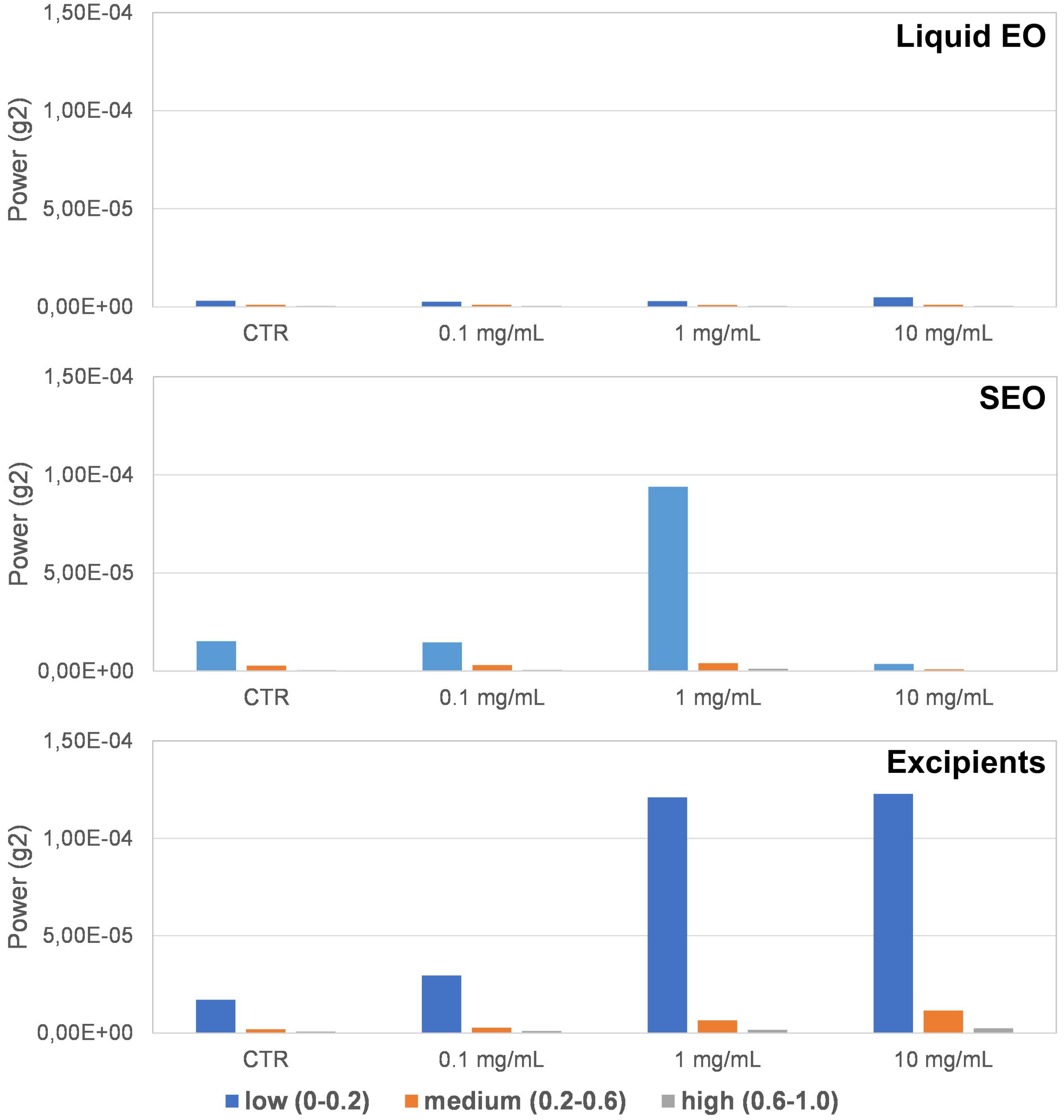

Figure S8. Effects of EO, SEO, excipients and OB on gastric fundus Spontaneous contraction rates (FFT). Absolute band powers of control and after addition of each concentration observed in the same experiment (FFT).

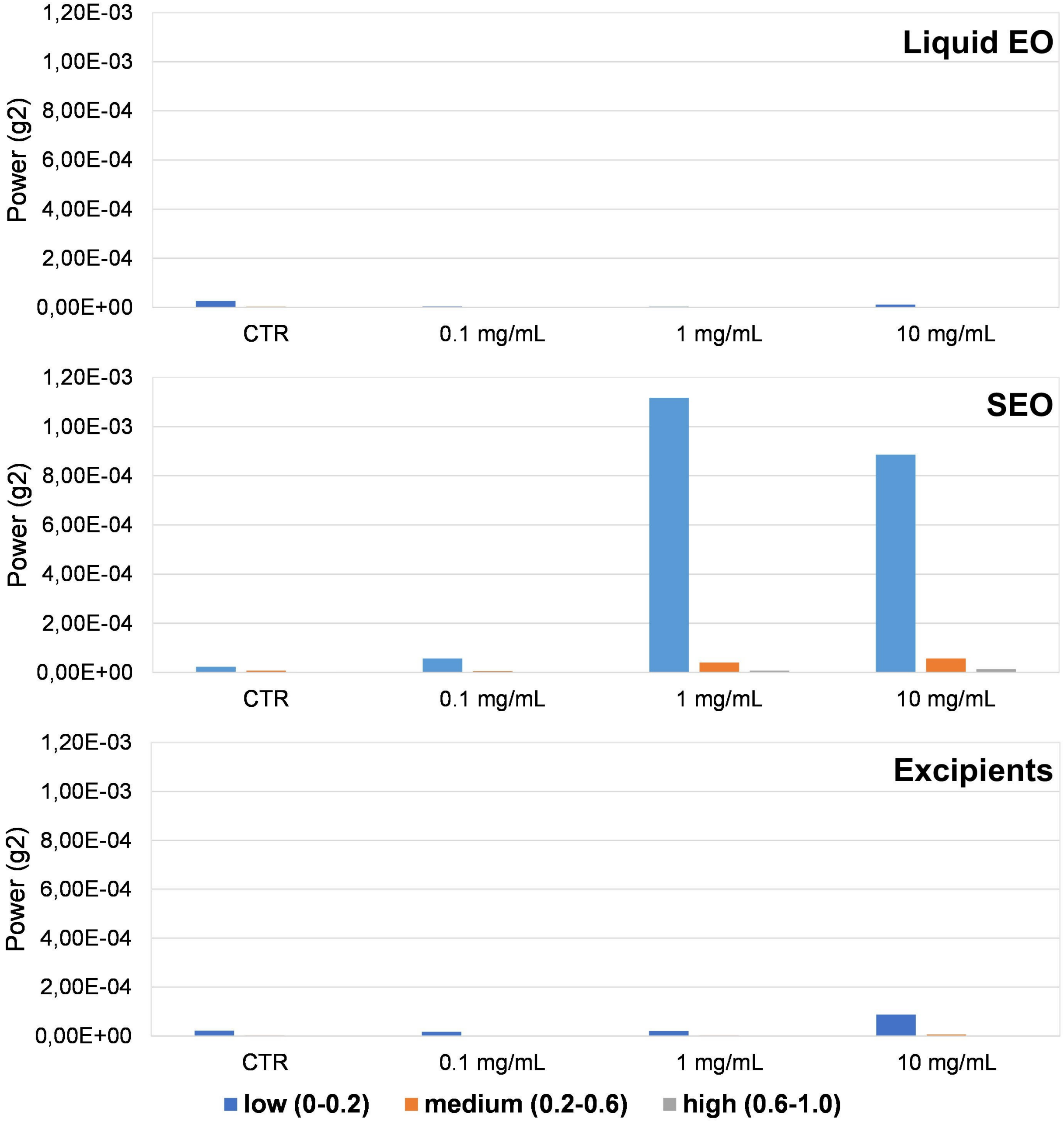

S9. Supplementary References

[SR1] Micucci, M.; Angeletti, A.; Cont M.; Corazza, I.; Aldini, R.; Donadio, E.; Chiarini, A.; Budriesi, R. Hibiscus flowers and olive leaves extracts based formulation for hypertension care: in vitro efficacy and toxicological profile. *J. Med. Food*, **2016**, *19*, 504–512.

[SR2] Micucci, M.; Ioan, P.; Aldini, R.; Cevenini, M.; Alvisi, V.; Ruffilli, C.; Chiarini, A.; Budriesi, R. Castanea sativa mill. extract induces gallbladder contraction and sphincter of Oddi relaxation in guinea pig: A natural approach to biliary tract motility disorders. *J. Med. Food*, **2014**, *17*, 795–803.

[SR3] Swenson JM, George E, Killgore FC: Tenover, Antimicrobial Susceptibility Testing of Acinetobacter spp. by NCCLS Broth Microdilution and Disk Diffusion Methods. *J. Clin. Microbiol.*, **2004**, *42*, 5102–5108.

[SR4] Micucci M, Gotti R, Corazza I, Tocci G, Chiarini A, De Giorgio M, Camarda L, Frosini M, Marzetti C, Cevenini M, Budriesi R. Newer Insights Into the Antidiarrheal Effects of *Acacia Catechu* Willd. Extract in Guinea Pig. *J Med Food*, **2016**, *20*, 592-600.
